# The stimulation mechanism of students’ entrepreneurial intention in entrepreneurship course: A trait activation theory perspective

**DOI:** 10.3389/fpsyg.2022.1031435

**Published:** 2022-11-24

**Authors:** Jun Wu, Wenhao Pan, Shuqiu Chen, Baijun Deng

**Affiliations:** ^1^School of Innovation and Entrepreneurship, Guangzhou Panyu Polytechnic, Guangzhou, China; ^2^Programs’ Development Department-DBA Office, Montpellier Business School, Montpellier, France

**Keywords:** creativity, entrepreneurial intentions, flow experience, entrepreneurship education, trait activation theory

## Abstract

This study examines the extent to which flow experience inhibits/enhances the effects of students’ creativity on their entrepreneurial intentions. This study provides evidence to support the contention that flow experience moderates the relationship between creativity and entrepreneurial intention by reference to a field survey of 226 Chinese college students in six college classes. Adopted by a hierarchical regression, this study found that creativity has a significant positive impact on entrepreneurial behavior. Within the subdimensions of flow, Intrinsic work motivation and Work enjoyment plays a significant positive moderating role in the relationship between creativity and entrepreneurial behavior, while absorption does not have such moderating effect. These findings reveal the process and mechanism by which creativity affects entrepreneurial intention and the associated psychological contingency factors.

## Introduction

In Chinese universities, all students are required to receive education in innovation and entrepreneurship. Whether college students are willing to devote themselves to the task of practicing innovation and entrepreneurship has become an important indicator when examining the teaching effect of innovation and entrepreneurship courses. However, although all students have received innovation and entrepreneurship education, the proportion of students who engage in innovation and entrepreneurship remains very low. The proportion of individuals with undergraduate degrees who are self-employed is 1.6%, and the same proportion of individuals with vocational degrees is 3.4%, both of which are much lower than the 20% figures reported for developed countries (Mei et al., 2016).

Entrepreneurial intention is the basis on which individuals are able to engage in entrepreneurial activities. In recent years, many scholars have noted that individual creativity plays a role that cannot be ignored in the process of enhancing individuals’ levels of entrepreneurial intention (Zampetakis and Moustakis, 2006; Hamidi et al., 2008). Although the relationship between individual creativity and entrepreneurial intention has been widely studied, extant research results remain not unanimous. For example, and Zampetakis et al. (2011) and Feldman and Bolino (2000a) pointed out that individual creativity is strongly correlated with entrepreneurial intention because people with high creativity can maintain a positive attitude and high self-confidence in entrepreneurial activities. However, Kumar and Shukla (2022) pointed out the direct effect of creativity on intention is very marginal in an empirical study. It indicated that the internal mechanism by which individual creativity affects entrepreneurial intention remains relatively vague and deserves further research. For example, we noticed that among the students who exhibited higher creativity scores on a preclass personal innovation trait test, only some of these students actively participated in entrepreneurial activities, while the other students displayed no strong interest in entrepreneurial activities. This situation causes us to wonder how the level of creativity exhibited by students affects their willingness to become entrepreneurs. What are the conditions and mechanisms underlying this effect? These issues have been investigated by educators and researchers.

Trait activation theory notes that when an individual possesses a certain personality trait, it is not necessarily the case that this trait will be activated and the individual will exhibit the associated behavior (Tett and Guterman, 2000). If the situation features stimuli that facilitate the expression of the trait, the effect of the trait will be amplified; otherwise, the trait will be inhibited. Whether creativity, as an individual trait, can be expressed effectively is determined by contextual factors (Tett and Burnett, 2003).

We note that psychological factors are a key contextual factor associated with creative expression. In recent years, an increasing number of scholars have realized the importance of combining research in the field of positive psychology with research concerning entrepreneurship. For example, empirical evidence has been found to suggest that entrepreneurial passion plays an important role in promoting entrepreneurial willingness, entrepreneurial effort, and entrepreneurial persistence in the context of entrepreneurial psychology and behavior (De Clercq et al., 2012). This finding shows that the individual’s psychological state plays a nonnegligible role in the implementation of entrepreneurial intentions and related actions. The theory of positive psychology highlights an important concept, i.e., that of “flow experience.” Csikszentmihalyi (1975) found that people may experience a unique form of flow experience in certain states, i.e., they feel sleepless, experience enjoyment, and seem to be bursting with amazing creativity. We also found that many students who will participate actively in entrepreneurial activities in the future exhibit active exploration and participate wholeheartedly in their innovation and entrepreneurship classes.

Therefore, this study attempts to employ the theoretical perspectives of trait activation theory and positive psychology to examine the facilitating/inhibitory effect of flow experience in specific situations on the process by which individual creativity affects entrepreneurial intention. Since the beginning of the COVID-19 pandemic, many courses taught in colleges and universities have shifted to online teaching, so we also tracked the status of students’ online courses. This study investigate a variety of data concerning 226 students in six classes in a Guangdong university pertaining to an online course on innovation and entrepreneurship, including students’ individual creativity, the degree of flow experience in the process of developing innovation and entrepreneurship projects in an online class, and the entrepreneurial intention exhibited by students after the class. We attempt to explain the process and mechanism by which creativity affects entrepreneurial intention and the associated psychological contingency factors.

This paper makes the following contributions to the literature. First, it enriches research concerning the relationship between creativity and entrepreneurial intention. Second, we examine the contingent effects of the three dimensions of flow experience on the main effects. Third, the concept of flow experience in positive psychology and research concerning the mechanism by which entrepreneurial behavior exerts its influence in the context of management are integrated into innovation and entrepreneurship education in colleges and universities, thus providing a new path for exploring college innovation and entrepreneurship education.

## Literature review and hypothesis development

### Creativity and entrepreneurship intentions

Creativity refers to an individual’s ability to recombine resources, information, and knowledge to generate novel, valuable ideas and his ability to use those ideas to create new careers (Amabile, 1996; Chua and Iyengar, 2008). High levels of creativity can help entrepreneurs make better judgments regarding the connections among resources, information and knowledge; identify business opportunities; overcome various difficulties in the entrepreneurial process, such as resource shortages and customer distrust, by generating creative solutions; and successfully start a new business and create new value (Biraglia and Kadile, 2017). As a result, creative individuals generate more ideas for products or services and have more confidence in engaging in entrepreneurship (McMullan and Kenworthy, 2015), thereby increasing their willingness to become entrepreneurs. This relationship has been verified by many empirical studies. For example, Zampetakis and Moustakis (2006) conducted a survey of 181 college students, and empirical analysis found that the more creativity the college students exhibited, the stronger their entrepreneurial intentions. A study by Zampetakis et al. (2011) further verified the relationship between these two factors. Hamidi et al. (2008) conducted a group comparison of 78 college students, and the results showed that the higher the scores received by college students on a creativity test were, the stronger their intention to start a business in the future. Both Feldman and Bolino (2000b) and Sternberg and Lubart (1999) empirically demonstrated that individuals who exhibit high creativity are more likely to open their own businesses.

Scholars have provided different explanations for the mechanism by which creativity affects entrepreneurial intention. From a personal trait perspective, Ardichvili et al. (2003) regarded creativity as an important personal trait and claimed that it can enhance people’s alertness to opportunities, enhance their ability to perceive opportunities around them, and promote their ability to identify entrepreneurial opportunities, thereby enhancing their entrepreneurial intentions. From a cognitive perspective, creative individuals form their own cognitive structures and related concepts *via* continuous learning, observation, and the accumulation of information (Biraglia and Kadile, 2017), and they generate innovative solutions *via* the continuous reorganization of knowledge and resources, subsequently allowing them to identify profitable entrepreneurial opportunities and thus enhancing their entrepreneurial intention.

In summary, creative individuals are better able to grasp and creatively use resources, information and knowledge, to discover and make full use of entrepreneurial opportunities, and to stimulate strong entrepreneurial intentions. Accordingly, this paper proposes the following hypothesis:

*H1*: Creativity has a positive effect on entrepreneurial intention.

### The moderating effect of flow experience

Many studies have shown that high levels of creativity are conducive to stimulating the entrepreneurial intention of individuals. However, the studies mentioned above have failed to explain the differences in entrepreneurial intention among highly creative individuals.

According to trait activation theory, whether individual traits can be expressed effectively is determined by many situational factors. Only when certain trait-related situational cues appear do these situational cues stimulate and exert pressure on the individual, and in these cases the underlying personality trait may be awakened and activated, which in turn encourages the individual to exhibit related behaviors (Tett and Burnett, 2003). Creativity, as a stable individual trait, may be more likely to stimulate entrepreneurial willingness in specific situations.

We focus on flow, a concept from positive psychology. Csikszentmihalyi (1975) studied human creativity and found that people may experience a unique experience when performing work that they love: forgetting sleep and food, forgetting everything else, and surrendering themselves to the work without receiving any reward. Individuals who have this experience often experience astonishing bursts of creativity and extremely high levels of innovative performance (Landhuer and Keller, 2012). Although flow experience is a mental state rather than an external situational factor as discussed by trait activation theory, it reflects an individual’s feelings regarding external situations and can thus be used as a representative variable for external situational cues. Bakker (2005) proposed the concept of work-related flow and claimed that the flow experience of individuals during the work process mainly includes three aspects: absorption, work enjoyment and intrinsic work motivation. This construct is used mainly to measure people’s mental states in the workplace. However, the situation in which students develop innovation and entrepreneurship projects in the innovation and entrepreneurship course is quite similar to the situation of the workplace. Therefore, we reference this context here.

Absorption is a state of intense concentration, in which the individual is so engrossed in his work that he forgets everything else around him. When an individual uses creativity to think innovatively, he may be thinking of new ways to solve problems or create new things. This process is certain to encounter many difficulties, tremendous challenges and substantial uncertainties. When the individual begins to concentrate his attention and when he invests all his energy into a certain matter and eliminates other distracting thoughts, this shift allows him to exercise cognitive and analytical abilities beyond their usual levels. This strengthening of these abilities can help individuals solve problems that cannot be solved in ordinary situations and successfully challenge difficulties that they would not dare to confront in ordinary times. Successfully dealing with difficult situations can cause individuals to experience positive emotions such as a sense of achievement and work pleasure (Staw et al., 1994). These emotions, in turn, help individuals continue to exercise their creativity to overcome difficulties and meet challenges (Csikszentmihalyi, 1997; Ryan and Deci, 2000). Therefore, when an individual uses creativity to discover entrepreneurial opportunities, the corresponding sense of focus strengthens the individual’s ability to act and his confidence in facing difficulties, thus making it easier for him to generate entrepreneurial intentions. Accordingly, this paper proposes the following hypothesis:

*H2*: Absorption moderates the relationship between creativity and entrepreneurial intention. The more absorption is the individual exhibits, the stronger the positive relationship between creativity and entrepreneurial intention.

The second dimension of flow is work enjoyment. Employees who enjoy their work and feel happy have very positive opinions regarding the quality of their work life. When an individual identifies an opportunity to engage in innovation and entrepreneurship through the exercise of creativity, that individual must confront a market environment that is full of uncertainty and fierce market competition, which is undoubtedly a tremendous challenge for individuals who have not yet entered the initial stage of entrepreneurship. If the individual attains the level of flow experience and especially a sense of work enjoyment when working on entrepreneurship projects during the innovation and entrepreneurship course, this situation can inspire strong positive emotions and a sense of optimism. Work enjoyment can have several effects on the formation of individual entrepreneurial intentions. First, individuals who experience a sense of enjoyment during the process of innovation and entrepreneurship education exhibit more optimistic judgments regarding various topics and are more tolerant of and can even generate new things and new ideas. Second, individuals who have a strong sense of well-being gain psychological capital, exhibit optimism, self-confidence and resilience, and show strong anti-frustration abilities in the face of difficulties (Harris et al., 2003). In addition, the general mentalities of employees who enjoy work are more positive, which can effectively alleviate the psychological pressure placed on individuals by the potential risks of their decision to start a business, thus prompting them to pursue this opportunity bravely. Accordingly, this paper proposes the following hypothesis:

*H3*: Work enjoyment moderates the relationship between creativity and entrepreneurial intention. The positive relationship between creativity and entrepreneurial intention is enhanced when individuals experience high levels of work enjoyment.

The third dimension of flow experience is intrinsic work motivation. Intrinsic work motivation refers to the inner experience of pleasure and satisfaction at work, which drives individuals to continue to be interested and engaged in their work (Bakker, 2008). Intrinsic motivation is the ideal motivational state for encouraging motivated members to exhibit positive behaviors (Ryan and Deci, 2000); in the context of intrinsic motivation, individuals feel interested in specific activities and can enjoy them and thus obtain inner satisfaction. Demerouti (2006) found that people who exhibit strong intrinsic motivation are committed to the pursuit of clear goals, and so they are more likely to engage in positive behaviors in the organization, such as pursuing goals and exhibiting the courage to challenge the status quo. On the other hand, flow experience and positive emotions are two-in-one concepts. Individuals form personal positive emotions due to their high intrinsic motivation to work toward and focus on their goals, thereby strengthening their cognitive functions (Staw et al., 1994). In other words, when individuals have high levels of intrinsic motivation, they are more optimistic and persevere in the face of difficulties and risks (Schaufeli and Bakker, 2004). This positive attitude, on the one hand, allows them to overcome all kinds of psychological obstacles to starting a business and, on the other hand, to confront possible failures and losses optimistically. The entrepreneurial opportunities that they highlight define their entrepreneurial willingness. Accordingly, this paper proposes the following hypothesis:

*H4*: Intrinsic motivation plays a moderating role in the relationship between creativity and entrepreneurial behavior. When intrinsic motivation is high, the positive relationship between creativity and entrepreneurial intention is enhanced.

**Figure 1:**
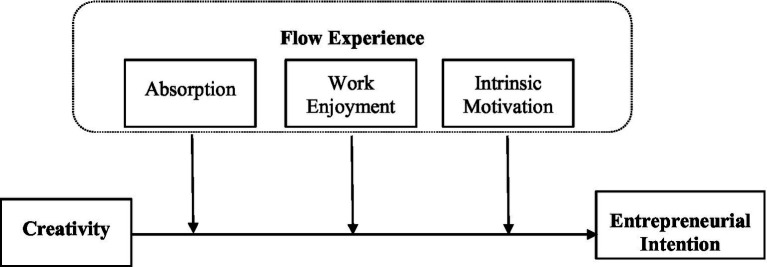
Research framework.

In summary, we develop a novel framework to examine the effects of creativity on entrepreneurial intentions with a particular focus on the moderating role played by flow experience in this context (see Figure 1).

## Methods and data

### Sample and data collection

In accordance with our research objectives, we took college students at a vocational university in China as our sample, selecting a total of 226 students from six classes in a science college and a literature college. The six classes were selected from classes taught by members of the research team, with the aim of ensuring a balance between engineering and liberal arts, between males and females, and between freshmen and sophomores.

Although convenience sampling has certain limitations, this approach is suitable for studying problems associated with small individual differences such as psychological mechanisms and psychological processes, those for which the general population is unknown, and those for which random sampling is very difficult. Students taught by the researchers were selected as the research object, which offered the possibility of obtaining real and effective data. Naturally, the use of non-probability sampling samples to infer some descriptive statistical characteristics of the population can often be biased, even systematically biased. However, previous research has shown that if a causal relationship is not moderated by group differences, the problem of sampling does not interfere with the ability to draw causal inferences (Druckman and Kam, 2011). In other words, if causality is universal, we should be able to observe the expected effect in a sample obtained by non-probability sampling. Many important theories in social science and psychology have also been studied and verified by reference to a small sample recruited through convenience sampling. Therefore, making causal inferences based on survey data recruited through non-probability sampling is accepted by the academic community in most cases (Kam et al., 2007).

The teacher explained the purpose of this research prior to administering the questionnaire and promised to use the resulting data only for academic purposes. In the investigation, the teacher did not guide the interviewed students to avoid subjective bias and provided sufficient time for the students to complete the questionnaire. All the interviewed students submitted their responses voluntarily.

We ultimately obtained a valid sample of 226 students. Table 1 presents the basic characteristics of the research sample. Among all respondents, 46% were male and 54% were female. Engineering majors accounted for 51.3% of the total, of whom 36 were architectural engineering students and 80 were municipal administration students. Liberal arts majors accounted for 48.7% of the total, of whom 82 were in the investment class and 28 were in the finance class.

**Table 1:** Characteristics of the research sample (*N* = 226).

		Frequency	Percentage
Gender	Male	104	46.0
	Female	122	54.0
Class	Architectural engineering	36	15.9
	Municipal administration	80	35.4
	Investment	82	36.3
	Finance	28	12.4

The possibility of nonresponse bias was addressed by comparing the gender and class of early and late respondents. This approach is consistent with the procedure proposed by Armstrong and Overton (1977), who argued that late respondents are more likely to resemble non-respondents than early respondents. The 143 respondents who completed the survey during the early stage were considered to be early respondents, while the 83 respondents who completed the survey in the late stage were considered to be late respondents. Chi-square tests of the early and late respondents indicated no significant differences in terms of gender (*p* > 0.05). Therefore, we ruled out the possibility of nonresponse bias.

We controlled for the effects of a single unmeasured latent method factor to test for the common method bias problem and used Mplus 8.3 software for statistical operations. As shown in Table 2, after unmeasured control factors were added, the fitting parameters of the model improved overall, but compared with the benchmark model, this improvement was small; the TLI and CFI scores increased by 0.026 and 0.025, while the SRMR and RMSEA distributions were reduced by 0.02 and 0.018, all of which were within a reasonable range. These findings indicate that the common methodological bias of this study was within the acceptable range.

**Table 2:** Common method bias test.

Model	*χ* ^2^	df	TLI	CFI	SRMR	RMSEA
Control model	297.158	158	0.961	0.97	0.032	0.062
Benchmark model	438.589	179	0.935	0.945	0.052	0.08
Improvement	/	/	0.026	0.025	0.02	0.018

TLI, Tucker–Lewis Index; CFI, comparative fit index; RMSEA, root mean square error of approximation.

### Measurement

First, based on the research purpose, we consulted the conventional literature in relevant fields published in the West extensively, ensured comprehensive induction and screening among existing questionnaire indicators and scales, and selected the most suitable scale for use in this research. Second, the first translation was produced by two members of the research team with doctoral degrees who had a deep understanding of the field, and the most appropriate translation of each measure of the scale was discussed jointly by these members. Subsequently, the other two members back-translated the translated Chinese measures into English simultaneously, and if the new English version of the sentence was found to differ significantly from the original English scale, they discussed the translation with each other and revised the Chinese translation. After implementing this process, the Chinese translation of the scale was finalized. Each item in the questionnaire was measured on a 5-point Likert scale (1 = strongly disagree; 5 = strongly agree), and the respondents were asked to evaluate the degree to which the statement conformed with their actual situation.

We adopted the alternative creativity scale adapted by Khedhaouria et al. (2015), which consists of four items. The scale was originally developed by Tierney et al. (1999) to include nine items. We retained Khedhaouria’s four items.

Entrepreneurial intention was operationalized as a construct featuring five formative dimensions (Mei et al., 2016, 2017). In a research article by this scholar, the scales were slightly different. We selected the test items based on different time configurations of entrepreneurial intention, that is, an uncertain schedule, an unlimited schedule, a limited schedule, and a definite schedule. We removed options that could not be measured using a Likert scale.

To measure flow, we employed three subdimensions of Bakker’s (2008) scale encompassing mental states related to absorption (four items), work enjoyment (four items), and intrinsic work motivation (five items). These dimensions can vary independently of one another. In our research, the dimensions vary independently, indicating a unique aspect of the flow construct.

## Results

### Descriptive analysis

We used SPSS 25.0 statistical software to conduct descriptive statistical analysis of the variables. As shown in Table 3, this study involved five variables; the minimum value of each variable was 1, the maximum value was 5, there were no outliers, and the mean values were all in a reasonable range. The sample number of the variables included in this study was 226, and so there were no missing values in this study. In addition, the skewness and kurtosis of each variable were <2, thus indicating that each variable was in accordance with a normal distribution.

**Table 3:** Descriptive analysis of five variables.

	*N*	Min	Max	Mean	Skewness	Kurtosis
Absorption	226	1	5	3.5022	−0.235	−0.408
Work enjoyment	226	1	5	4.0166	−1.086	1.085
Intrinsic work motivation	226	1	5	3.8389	−0.884	0.881
Creativity	226	1	5	3.5542	−0.188	−0.255
Entrepreneurial intention	226	1	5	3.4336	−0.293	−0.079

### Reliability and validity

We used Mplus 8.3 software to examine construct reliability and validity. Table 4 displays the descriptive statistics for the study, including the Cronbach’s *α* coefficients, loading, composite reliability, and average variance extracted of all 21 variables. All constructs’ Cronbach’s *α* coefficients exceeded the suggested minimum of 0.70, indicating sufficient reliability (Cronbach, 1951; Nunnally, 1978; Muthén and Muthén, 2007).

**Table 4:** Construct measurements, reliability and validity.

Constructs and measurement	Cronbach’s *α*	Loading	C.R.	AVE
FLOW – Absorption	0.884		0.891	0.673
Absorption 1		0.771		
Absorption 2		0.716		
Absorption 3		0.837		
Absorption 4		0.941		
FLOW – Work enjoyment	0.961		0.962	0.864
Work Enjoyment 1		0.874		
Work Enjoyment 2		0.941		
Work Enjoyment 3		0.961		
Work Enjoyment 4		0.939		
FLOW – Intrinsic work motivation	0.914		0.916	0.687
Intrinsic motivation 1		0.907		
Intrinsic motivation 2		0.727		
Intrinsic motivation 3		0.893		
Intrinsic motivation 4		0.760		
Intrinsic motivation 5		0.843		
Creativity	0.914		0.915	0.730
Creativity 1		0.790		
Creativity 2		0.869		
Creativity 3		0.865		
Creativity 4		0.890		
Entrepreneurial intention	0.901		0.903	0.699
Entrepreneurial intention 1		0.892		
Entrepreneurial intention 2		0.822		
Entrepreneurial intention 3		0.852		
Entrepreneurial intention 4		0.775		

### Hypotheses testing

To examine the main effect of the relationship between creativity and entrepreneurial intentions as well as the moderating effects of the three dimensions of flow—absorption, work enjoyment, and intrinsic work motivation—we used the SPSS 25.0 statistical software to conduct hierarchical regression analysis on the main effect and moderating effects. We further used PROCESS Procedure for SPSS Version 4.0 to account for moderating effects; the VI*F* value of each independent variable regression on the dependent variable was <5. Based on our research hypotheses, we referred to the test method developed by Hayes and Matthes (2009), Hayes (2013), and Hayes and Agler (2014) to examine the moderating effects. If the regression coefficient of the interaction term between the independent variable and the moderator variable is significant, a moderating effect is indicated; otherwise, no moderating effect is indicated. Considering the problem of collinearity, the independent variables and moderator variables were standardized before the analysis was conducted. The results are shown in the Tables 5–7.

**Table 5:** Moderating effect analysis (flow – absorption; *N* = 226).

	Model 1	Model 2	Model 3
Constant	3.434** (72.528)	3.434** (74.746)	3.401** (63.473)
Creativity	0.750** (13.707)	0.556** (7.608)	0.559** (7.650)
Absorption		0.279** (3.862)	0.286** (3.947)
Creativity * Absorption			0.063 (1.182)
*R* ^2^	0.456	0.49	0.493
Adjusted *R*^2^	0.454	0.486	0.487
*F*-value	*F* = 187.882, p = 0.000	*F* = 107.232, *p* = 0.000	*F* = 72.081, p = 0.000
Δ*R*^2^	0.456	0.034	0.003
Δ*F*-value	F = 187.882, *p* = 0.000	*F* = 14.913, p = 0.000	*F* = 1.397, *p* = 0.238
VIF	1	1.864, 1.864	1.867, 1.868, 1.014
Dependent Variable: Entrepreneurial Intention			

The *t*-value is included in parentheses.

***p < 0.001; **p < 0.01; *p < 0.05; +*p* < 0.1.

**Table 6:** Moderating effect analysis (flow – work enjoyment; *N* = 226).

	Model 1	Model 4	Model 5
Constant	3.434** (72.528)	3.434** (73.321)	3.389** (63.344)
Creativity	0.750** (13.707)	0.636** (8.895)	0.619** (8.598)
Work enjoyment		0.163* (2.434)	0.222** (2.949)
Creativity * Work enjoyment			0.085^+^ (1.695)
*R* ^2^	0.456	0.47	0.477
Adjusted *R*^2^	0.454	0.465	0.47
*F*-value	*F* = 187.882, *p* = 0.000	*F* = 98.968, *p* = 0.000	*F* = 67.490, *p* = 0.000
Δ*R*^2^	0.456	0.014	0.007
Δ*F*-value	*F* = 187.882, *p* = 0.000	*F* = 5.924, *p* = 0.016	*F* = 2.872, *p* = 0.092
VIF	1	1.746, 1.746	1.783, 2.212, 1.332
Dependent Variable: Entrepreneurial Intention			

The *t*-value is included in parentheses.

***p < 0.001; **p < 0.01; *p < 0.05; +*p* < 0.1.

**Table 7:** Moderating effect analysis (intrinsic work motivation; *N* = 226).

	Model 1	Model 6	Model 7
Constant	3.434** (72.528)	3.434** (74.769)	3.368** (63.969)
Creativity	0.750** (13.707)	0.521** (6.558)	0.491** (6.178)
Intrinsic work motivation		0.311** (3.880)	0.384** (4.537)
Creativity * Intrinsic work motivation			0.119* (2.461)
*R* ^2^	0.456	0.491	0.504
Adjusted *R*^2^	0.454	0.486	0.497
*F*-value	*F* = 187.882, *p* = 0.000	*F* = 107.361, *p* = 0.000	*F* = 75.216, *p* = 0.000
Δ*R*^2^	0.456	0.034	0.014
Δ*F*-value	*F* = 187.882, *p* = 0.000	*F* = 15.054, *p* = 0.000	*F* = 6.056, *p* = 0.015
VIF	1	2.239, 2.239	2.292, 2.551, 1.173
Dependent Variable: Entrepreneurial Intention			

***p < 0.001; **p < 0.01; *p < 0.05; +*p* < 0.1.

The *t*-value is included in parentheses.

As these tables indicate, in each model of the moderating effect, the interaction terms among the independent variables, work enjoyment and intrinsic work motivation were significant, indicating that there was a moderating effect. The interaction term between the independent variable and absorption was not significant, indicating that there was no moderating effect. Moreover, according to the positive and negative signs of the interaction term, work enjoyment and intrinsic work motivation play important positive moderating roles in the process by which the independent variable affects the dependent variable; that is, when work enjoyment or intrinsic work motivation is high, the degree to which creativity influences entrepreneurial intention increases significantly. Therefore, Hypotheses 1, 3 and 4 were supported, while Hypothesis 2 was not supported.

**Figure 2:**
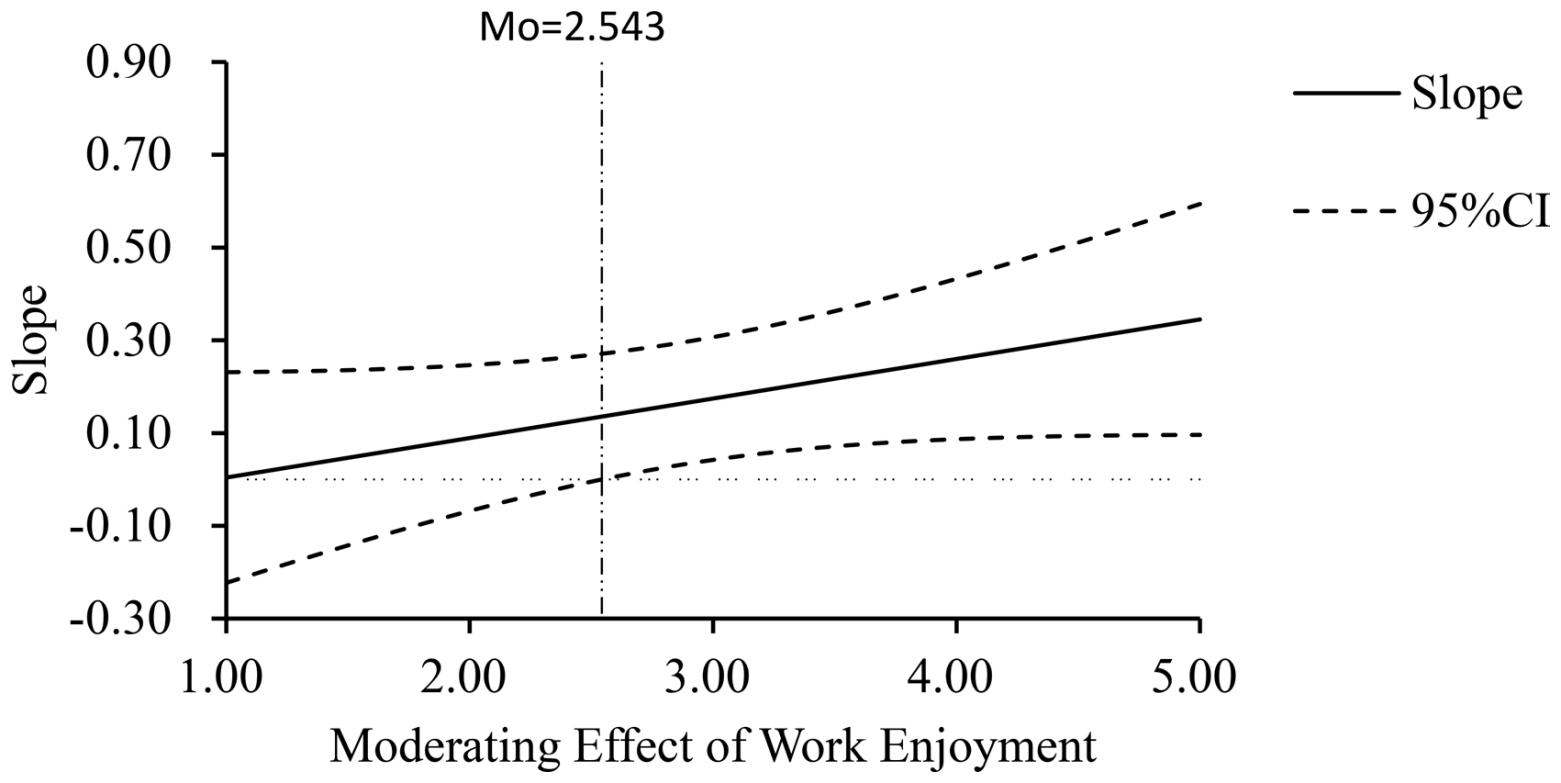
The moderating effect of work enjoyment (Johnson–Neyman method).

The Johnson–Neyman Technique (Johnson and Neyman, 1936) can display the transition node of the simple slope from significant to nonsignificant when the moderating variable takes different values. This approach helps to explore the value range of the moderating variable, and the corresponding simple slope is significant. As shown in Figure 2, the critical value of work enjoyment is 2.543, indicating that when work enjoyment is >2.543, there is a significant moderating effect.

**Figure 3:**
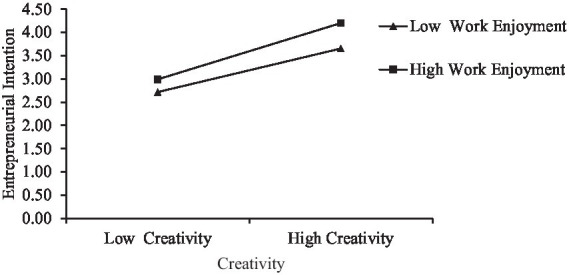
The slope of the moderating effect of work enjoyment.

Simple slope analysis can demonstrate the moderating interaction visually. As shown in Figure 3, work enjoyment has a positive impact on entrepreneurial intention. In the case of high work enjoyment, creativity has a greater impact on entrepreneurial intention, thus indicating that work enjoyment can strengthen or promote the relationship between these factors. The moderating effect of Hypothesis 3 is tested once again.

**Figure 4:**
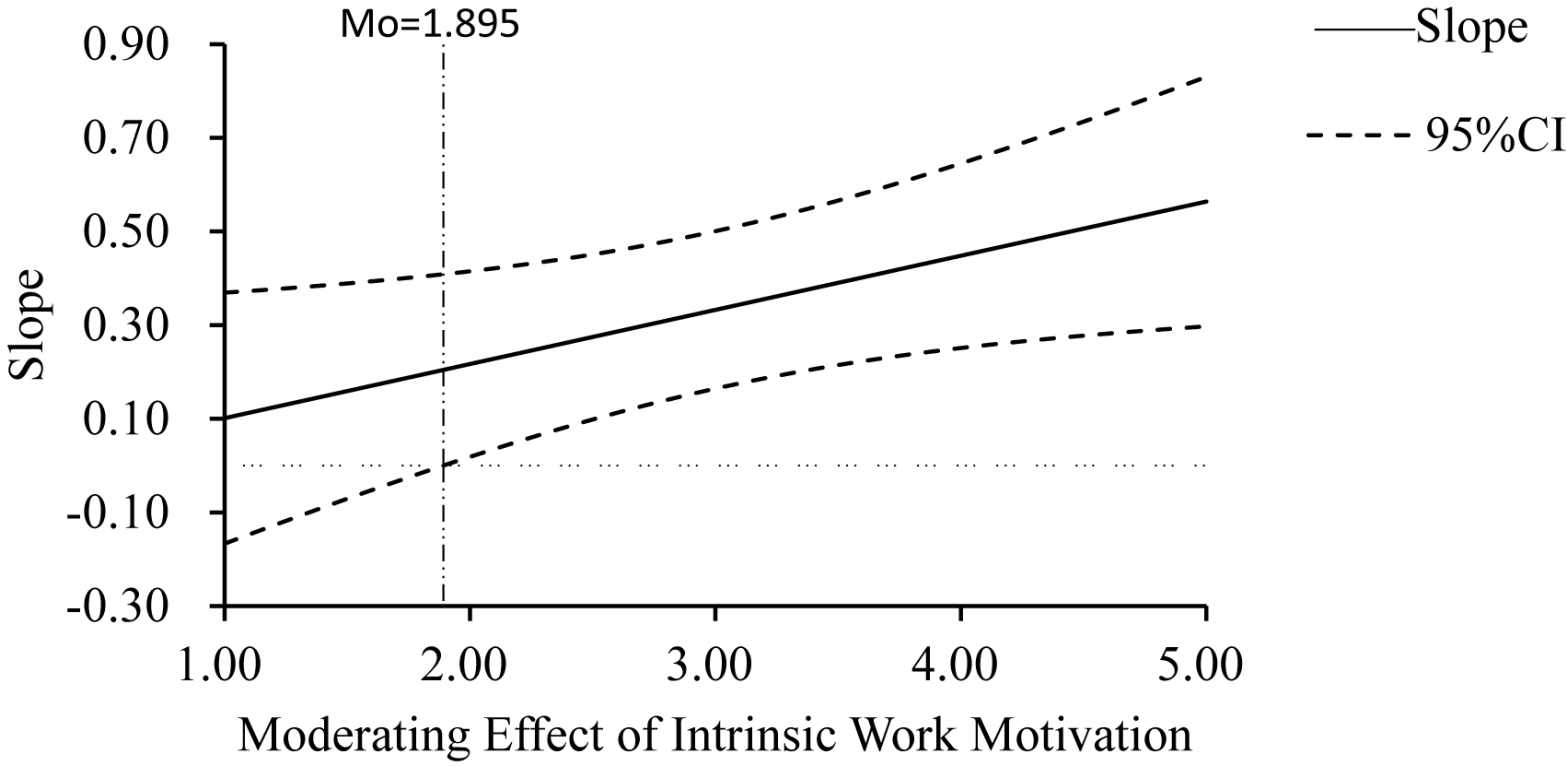
The moderating effect of intrinsic work motivation (Johnson–Neyman method).

As shown in Figure 4, the critical value of intrinsic work motivation is 1.895, thus indicating that when intrinsic work motivation is >1.895, there is a significant moderating effect.

**Figure 5:**
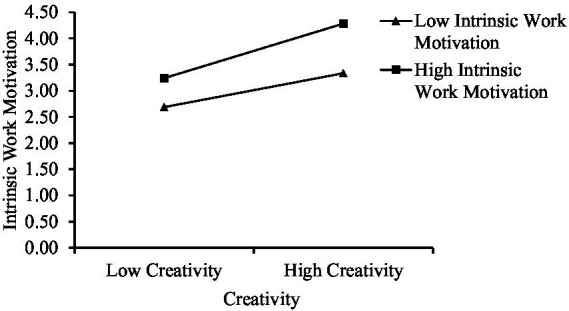
The slope of the moderating effect of intrinsic work motivation.

As shown in Figure 5, intrinsic work motivation has a positive impact on entrepreneurial intention. In the case of high intrinsic work motivation, creativity has a greater impact on entrepreneurial intention, thus indicating that intrinsic work motivation can strengthen or promote the relationship between these factors. The moderating effect of Hypothesis 4 is tested once again.

## Discussion

### Discussion of results

This study explores the extent to which flow experience inhibits/enhances the impact of student creativity on entrepreneurial behavior. By reference to a questionnaire survey of 226 college students in six classes in a higher vocational college in China, the empirical results of the study support two of the hypotheses proposed in this paper. The main conclusions of this study are as follows:

1. Creativity has a significant positive impact on entrepreneurial behavior.

The research results show that the more creativity college students exhibit, the more entrepreneurial behaviors are stimulated. This conclusion supports the claims that colleges and universities can cultivate students’ creativity by shaping the curriculum and providing comprehensive quality training and that they can then influence students to participate actively in entrepreneurship and stimulate students’ enthusiasm for entrepreneurship.

2. Intrinsic work motivation plays a significant positive moderating role in the relationship between creativity and entrepreneurial behavior.

The results show that creativity has a stronger positive effect on entrepreneurial behavior in the context of strong intrinsic work motivation; that is, strong intrinsic work motivation enhances the positive effect of creativity on entrepreneurial behavior. This conclusion reveals that the main effect of flow experience is to strengthen the positive impact of creativity on entrepreneurial behavior by enhancing students’ intrinsic work motivation. This conclusion supports the claim that colleges and universities can guide students to immerse themselves in entrepreneurial activities by cultivating students’ creativity and creating a high-flow experience environment in the course so that students can engage in more entrepreneurial behaviors and participate actively in entrepreneurship.

3. Work enjoyment has a positive moderating effect on the relationship between creativity and entrepreneurial intention.

The results of this study indicate that when students feel work enjoyment when completing their classroom tasks, the effect of creativity on entrepreneurial intention is increased. This conclusion supports the claim that when preparing for entrepreneurship courses, teachers should design entrepreneurial tasks that are moderately difficult, which can allow students to enjoy the process of completing entrepreneurial tasks. This approach can encourage students to express their creativity to a greater extent, thereby enhancing their willingness to become entrepreneurs.

4. Absorption does not have a moderating effect on the relationship between creativity and entrepreneurial intention.

With respect to the problem that the moderating effect of absorption is not significant, a possible explanation for this result is that students’ concentration on class does not affect students’ creativity and entrepreneurial willingness significantly. The changes in creativity and entrepreneurial intention may be more closely related to factors external to the course, such as knowledge, experience and talents. The research conducted by Shih and Huang (2017) indicated that students’ post-course understanding has a significant effect on students’ entrepreneurial intentions. Students’ learning and experiences outside the entrepreneurial course may lead to different understandings and thus cause students to exhibit different levels of entrepreneurial intention.

### Theoretical contributions

Previous studies have reached a general consensus that creativity is beneficial with respect to stimulating individual entrepreneurial intentions (Ardichvili et al., 2003; Zampetakis and Moustakis, 2006; Hamidi et al., 2008). However, previous studies have failed to explain the differences in entrepreneurial intentions among highly creative individuals. From the perspective of positive psychology, this study takes the flow experience as a starting point to investigate the innovation and entrepreneurship education of college students, highlights the feasibility of the practical application of flow experience in the context of innovation and entrepreneurship education in colleges and universities, and provides a basis for the construction of a classroom that is conducive to flow experience in the future. The research provides the associated theoretical foundation.

This study found that creativity has a significant positive impact on entrepreneurial behavior, thus echoing the research of scholars such as Zampetakis and Moustakis (2006) and confirming that this conclusion can be applied to the research of vocational students, thereby extending the scope of application of the conclusion. Furthermore, we explored the moderating roles played by three dimensions of flow experience, namely, focus, intrinsic work motivation, and work enjoyment, in the relationship between creativity and entrepreneurial intention. Based on the theoretical analysis and data verification discussed above, this paper integrates and enriches the literature concerning the relationship between creativity and entrepreneurial intention and makes the following three theoretical contributions.

First, this study enriches the research concerning the relationship between creativity and entrepreneurial intention. The conclusion of previous studies that creativity has a significant positive impact on entrepreneurial intention has received general agreement, but the moderating variables in this relationship have rarely been discussed. By introducing the concept of flow experience, this study theoretically enriches our understanding of the moderating mechanism operative in this relationship and provides a new perspective for studying the relationship between creativity and entrepreneurial intention. In addition, previous studies have mainly focused on college students as research objects, and vocational students and college students have different starting points and directions with respect to their training.

Second, we examine the contingent effects of the three dimensions of flow experience on the main effects. According to this study’s review of previous studies, creativity can drive individuals to engage in entrepreneurial behavior; accordingly, this research proposes and validates the hypothesis that creativity has a significant positive impact on entrepreneurial behavior. However, few studies have discussed the moderating mechanisms associated with the relationship between creativity and entrepreneurial behavior. Taking into account the relationship between psychology and behavior, this study introduces the concept of flow experience from positive psychology as a moderator into the relationship between creativity and entrepreneurial behavior in an attempt to explain the impact of entrepreneurial behavior in terms of a mechanism operating on the psychological level. The conclusions of this study suggest that the flow experience regulates the two perspectives of intrinsic work motivation and focus. By creating an environment conducive to entrepreneurial flow experience, students’ intrinsic work motivation and classroom learning task focus can be enhanced, thereby strengthening the positive impact of creativity on entrepreneurial intention. The conclusions of this study highlight the feasibility of using flow experience as a moderator variable and provide new ideas that can be used by related research concerning entrepreneurial behavior.

Third, this study integrates the concept of flow experience from positive psychology and research concerning the mechanism by which entrepreneurial behavior impacts management in the context of innovation and entrepreneurship education in colleges and universities. The integration of these three disciplines provides a new path for exploring innovation and entrepreneurship education in colleges and universities and suggests useful insights regarding methods of innovation and entrepreneurship education. This study found that by cultivating students’ creativity and creating a classroom environment that is conducive to flow experience, students can become motivated to engage in more entrepreneurial behaviors and generate more entrepreneurial achievements.

### Managerial implications

The innovation and entrepreneurship ability of college students refers to their awareness of and ability to produce innovation as well as their potential for entrepreneurship. From the perspective of cultivating talent in colleges and universities, the emphasis is on improving the comprehensive quality and abilities of students. From the perspective of society’s demand for talent, the target of examination is the practical ability of students. Therefore, whether college students are in school or entering society, innovation and entrepreneurship are indispensable.

1. Course content should keep pace with the times and be closely connected to students’ lives.

According to the conclusions of this study, intrinsic work motivation plays a significant positive moderating role in the relationship between creativity and entrepreneurial intention. If students can continue to express interest in classroom content and to be able to devote themselves to classroom learning tasks, these traits can enhance students’ entrepreneurial willingness.

When compiling a series of teaching materials to promote innovation and entrepreneurship, the cases referenced should be forward-looking, and the most recent hot topics should be included in the teaching materials. Students taking courses on innovation and entrepreneurship should not only master relevant theoretical knowledge but also be able to apply the knowledge they have acquired to guide entrepreneurial practice. When preparing lessons, teachers should ensure that the content of classroom teaching remains closely connected to students’ lives as well as their hobbies and living habits to stimulate students’ interest in learning and to render the knowledge that students acquire applicable to their own lives and practices.

2. Online teaching should be used in a flexible manner to strengthen interaction between teachers and students both inside and outside the classroom.

According to the conclusions of this study, focus plays a positive moderating role in the relationship between creativity and entrepreneurial intention. College teachers can construct a three-dimensional learning atmosphere to promote students’ innovation and entrepreneurship using multimedia, multiplatform, and multiangle teaching methods in the context of online and offline hybrid teaching, and they can integrate the learning tasks assigned in innovation and entrepreneurship courses into students’ lives to improve students’ ability to learn in innovation and entrepreneurship courses. This focus can help strengthen students’ entrepreneurial aspirations.

Some schools use online platforms such as Chaoxing Xuexitong and WeChat to construct online innovation and entrepreneurship education platforms. However, some schools use these platforms only to publish information and online learning materials, and students use the various learning platforms provided by the school only as sources of information. In fact, these learning platforms have many functions in addition to their ability to publish information. Teachers can use the online teaching platform to strengthen classroom interaction with students, assign classroom tasks, allow students to think independently or work in groups to accomplish learning goals, and ensure that students continue to think about the classroom content. For example, they can publish discussions of points related to class knowledge, online quizzes, homework assignments, group learning exercises and other materials. Teachers can use these functions to enrich online learning content so that students are able to engage in independent learning on the online platform after class. This approach can help students abandon the concept of “learning in the classroom and life outside the classroom,” integrate learning into their own lives, and enter a state of flow experience in the context of innovation and entrepreneurship learning.

### Limitations and directions for future research

Some of the studies included in this research remain insufficient and can be improved by follow-up research. First, this study focuses mainly on the moderating role of the three dimensions of flow experience in the relationship between creativity and entrepreneurial behavior. Many variables that have been studied widely in the field of psychology may be involved in this above relationship as mediating variables or adjustment variables. Future research can enrich our understanding of this topic and analyze the mediating and moderating effects of different variables on the relationship between creativity and entrepreneurial intention. Second, this study mainly takes vocational students as its research object and analyzes the positive moderating effect of flow experience on creativity and entrepreneurial intention. However, whether this conclusion can be extended to other research objects, such as colleges and enterprise employees, remains to be confirmed.

## Data availability statement

The raw data supporting the conclusions of this article will be made available by the authors, without undue reservation.

## Author contributions

JW led the research design and wrote the theory part. BD is responsible for data analysis. SC collected data and participated in the study of theory part. WP participated in the writing of data analysis and contribution. All authors contributed to the article and approved the submitted version.

## Funding

Research Project of Guangzhou Education Bureau (2019PT102, 2020KC018, 2022CXCYXY001, and 2022CXCYZX005), Guangzhou Polytechnic Research Project (2021CY05).

## Conflict of interest

The authors declare that the research was conducted in the absence of any commercial or financial relationships that could be construed as a potential conflict of interest.

## Publisher’s note

All claims expressed in this article are solely those of the authors and do not necessarily represent those of their affiliated organizations, or those of the publisher, the editors and the reviewers. Any product that may be evaluated in this article, or claim that may be made by its manufacturer, is not guaranteed or endorsed by the publisher.
